# Roles of peptidyl-prolyl isomerase Pin1 in disease pathogenesis

**DOI:** 10.7150/thno.45889

**Published:** 2021-01-19

**Authors:** Jingyi Li, Chunfen Mo, Yifan Guo, Bowen Zhang, Xiao Feng, Qiuyue Si, Xiaobo Wu, Zhe Zhao, Lixin Gong, Dan He, Jichun Shao

**Affiliations:** 1 The Second Affiliated Hospital of Chengdu Medical College, China National Nuclear Corporation 416 Hospital, Chengdu, Sichuan, China.; 2 School of Biological Sciences and Technology, Chengdu Medical College, Chengdu, China.; 3 Department of Immunology, School of Basic Medical Sciences, Chengdu Medical College, Chengdu, China.

**Keywords:** peptidyl-prolyl cis-trans isomerases, Pin1, pathogenesis of diseases, viral infection, neurodegenerative diseases

## Abstract

Pin1 belongs to the peptidyl-prolyl cis-trans isomerases (PPIases) superfamily and catalyzes the cis-trans conversion of proline in target substrates to modulate diverse cellular functions including cell cycle progression, cell motility, and apoptosis. Dysregulation of Pin1 has wide-ranging influences on the fate of cells; therefore, it is closely related to the occurrence and development of various diseases. This review summarizes the current knowledge of Pin1 in disease pathogenesis.

## Introduction

Some proteins exhibit either a cis or a trans state due to the presence of proline, which imparts distinct conformations and biological functions. The peptidyl-prolyl cis-trans isomerases (PPIases) superfamily 1 comprises four families according to their structural differences: cyclophilins, FK506-binding proteins (FKBPs), parvulins, and the protein phosphatase (PPase) 2A phosphatase activator (PTPA) 2-6.

In the human parvulin family, there are two genes: PIN1 and PIN4 7-9. The coded product of *PIN1*, PPIase NIMA-interacting 1 (Pin1) protein, was identified in 1996 by Lu *et al.* as a protein interacting with NIMA kinase 7. PIN4 encodes the isoforms parvulin 14 (Par14) and parvulin 17 (Par17) 9, 10. Parvulin 14 consists of 131 amino acids, while parvulin 17 is an N-terminal extended version of Par14 with an additional 25 amino acids. Among the three members of the human parvulin family, current research on the function of Pin1 and its role in disease pathogenesis is the most in-depth. Pin1 consists of 163 amino acid residues with a relative molecular mass of 18 kDa and contains 1 nuclear localization signal and 2 functional domains. The amino terminus (N-terminus) is the tryptophan-tryptophan central domain (WW domain), which is responsible for recognition and binding to the pSer/Thr-Pro motif of the substrate, while the C-terminal PPIase catalytic domain performs the function of cis-trans isomerization 7, 11. The two domains are fastened by a flexible linker of 15 residues. Although they belong to the same family, Pin1 differs from parvulin-type PPIases in that Pin1 specifically catalyzes the isomerization of phosphorylated Ser-Pro or Thr-Pro (pSer-Pro or pThr-Pro) peptides, whereas Par14/Par17 show no preference for phosphorylated substrates 9, 12, 13.

Since Pin1 isomerizes phosphorylated substrates and phosphorylation and post-phosphorylation events play important roles in cell signaling pathways, Pin1 is involved in a variety of cellular processes such as cell cycle, cell proliferation, cell motility, and apoptosis 13-18. In most cases, Pin1 functions as a molecular timer or switch that modulates proteins or entire signaling pathways. Dysregulation of Pin1 is closely related to the development of multiple diseases. In this review, we will discuss the role of Pin1 in the pathogenesis of various related diseases.

## Pin1 and cancer

**Figure 1:**
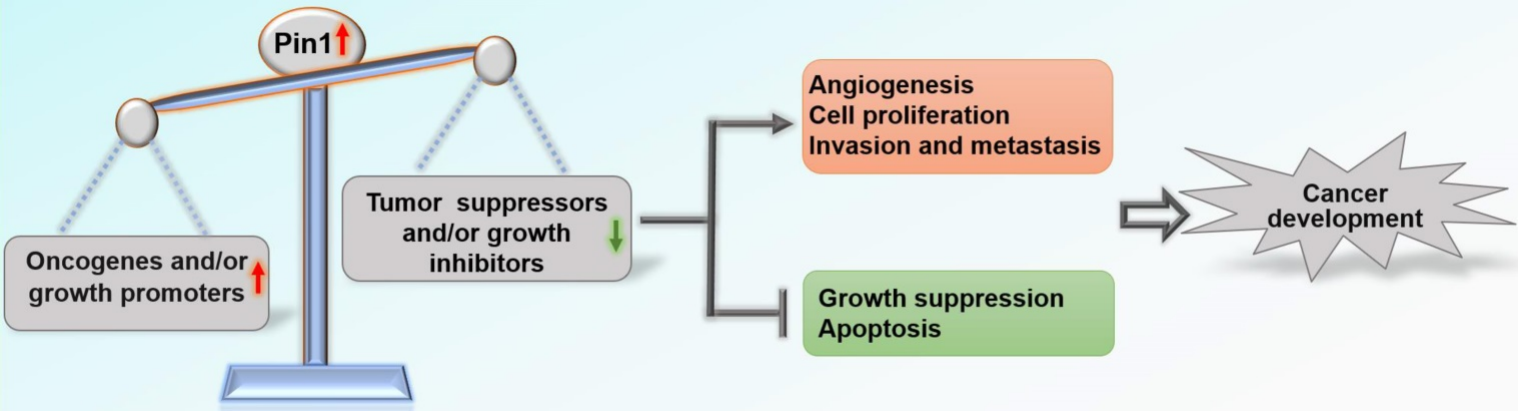
** Roles of Pin1 in cancer development.** Pin1 overactivation disrupts the balance between oncogenes and tumor suppressors, which affects biological behaviors related to tumor development.

Overall, Pin1 drives tumor progression and is negatively associated with clinical outcome in patients with cancer 19-21. Pin1 has been shown to activate more than 50 oncogenic proteins and growth promoters and/or shut down at least 20 tumor suppressors and growth inhibitors through positive and negative feedback mechanisms 12, 22 **(Table 1)**. Most tumors exhibit overexpression and/or activation of Pin1 compared with corresponding normal tissues, including breast, prostate, lung, ovarian, gastric, esophageal, cervical, and brain tumors and melanoma 21, 23-25. Expression of Pin1 in tumor cell lines cultured *in vitro* has also been found to be significantly higher than that in normal cell lines. Knockdown of the Pin1 gene inhibits cancer cell growth both *in vitro* and *in vivo* and results in cancer cell apoptosis 26, 27. In addition, emerging evidence suggests that inhibitors targeting Pin1 have significant anti-cancer effects. These inhibitors include juglone 27, 28, all-trans retinoic acid (ATRA) 29, 30, 2-{[4-(4-tert-butylbenzenesulfonamido) -1-oxo-1,4-dihydronaphthalen-2-yl] sulfanyl} acetic acid (KPT-6566) 31, epigallocatechin-3-gallate (EGCG) 32, PiB 33, compound 20 34, compound 23a 35, API-1 36, arsenic trioxide (ATO) 37, and BJP-06-005-3 38. In a recent review 22, Chen *et al.* elaborated on how Pin1 contributes to all ten hallmarks of cancer 39 by dysregulating multiple cancer-driving pathways at various levels. Pin1 induces angiogenesis by facilitating expression of VEGF and inhibition of Pin1 by RNAi significantly suppresses angiogenesis 40, 41; Pin1 sustains proliferative signaling and evades growth suppression by activating growth-promoting regulators and inactivating growth-inhibitory regulators 22; Pin1 promotes migration and invasion by regulating NOTCH1 42, TGF-β 43, β-catenin 44, and BRD4 45; Pin1 inhibits apoptosis of tumor cells by increasing the anti-apoptotic function of anti-apoptotic proteins and suppressing pro-apoptotic factors 46, 47. This concept has recently been greatly expanded, demonstrating that overactivation of Pin1 disrupts the balance between carcinogenic proteins and tumor suppressors, which pushes cells towards carcinogenesis 12 **(Figure 1)**.

## Pin1 and cardiovascular diseases

Atherosclerosis (AS) is a chronic disease and the main cause of coronary heart disease, cerebral infarction, and peripheral vascular disease 48. The early stage of AS is mainly caused by endothelial dysfunction. Endothelial nitric oxide synthetase (eNOS) plays a key role in the control of blood pressure and prevention of atherosclerosis by producing the vasodilator and vascular protective molecule nitric oxide (NO) 49. eNOS interacts with Pin1 in a phosphorylation-dependent manner in endothelial cells. Phosphorylation of eNOS at Ser^116^ enhances this interaction, thus inhibiting eNOS activity and reducing NO release 50, 51. Pin1 also drives diabetic vascular disease by causing mitochondrial oxidative stress and ROS production. Inhibition of Pin1 by gene silencing in human aortic endothelial cells (HAECs) or Pin1 knockout in mice was found to restore NO levels and relieve vascular dysfunction 52. These results indicate that Pin1 reduces NO synthesis by inhibiting eNOS and, thus, exerts a negative effect in cardiovascular disease.

However, in some conditions, Pin1 may protect vascular endothelial homeostasis. TGF-β stimulates synthesis of proteoglycan in vascular smooth muscle cells (VSMC), especially expression of disaccharide chain protein and extension of glycosaminoglycan (GAG) chain on biglycan, which increases lipoprotein binding and promotes early inflammation of atherosclerosis 53, 54. It has been shown that Pin1 enhances degradation of Smad2/3 ubiquitin proteasome induced by Smurf2 and inhibits TGF-β signal transduction 55, effectively preventing early occurrence of atherosclerosis 56. Another study showed that Pin1 inhibition significantly suppresses NO production in human periodontal ligament cells (PDLCs) 57. Taken together, Pin1 potentially plays a double-edged role in regulating the pathogenesis of cardiovascular diseases under different circumstances. Similarly, both overexpression and downregulation of Pin1 can reduce cardiac hypertrophy 58. Further detailed investigations are needed to reveal the function of Pin1 in cardiovascular disease.

## Pin1 and metabolic diseases

Insulin dysregulation is associated with various metabolic diseases including obesity, NASH, and type 2 diabetes. Pin1 promotes insulin secretion of islet β cells by enhancing the activity of SIK2, and also promotes cell proliferation and transformation by regulating activation of AP1 and ERK1/2 induced by insulin through interaction with p70S6K 59, 60. Pin1 also positively regulates insulin-induced phosphorylation of IRS-1: Pin1 deletion inactivates IRS-1, thus leading to insulin resistance 61. It can be concluded that Pin1 is involved in these metabolic diseases partially by controlling insulin signaling. However, Pin1 interacts with or regulates other key molecules involved in metabolic diseases, including obesity-related factors AMPK 62-65, PPARγ 66, and PRDM16 67; osteoporosis-related factors Runx2 68-70 and BMP2 71; and Nash-related factors Smad2/Smad3 and the TGF-β1 pathway 72. The detailed mechanisms by which Pin1 regulates metabolic diseases are summarized in other reviews 73, 74.

## Pin1 and neurodegenerative diseases

Although emerging evidence has shown that Pin1 directly or indirectly regulates neuronal proteins such as Tau, amyloid precursor protein (APP), and α-synuclein, the physiological functions of Pin1 in neurodegenerative diseases remain to be elucidated. For example, in Parkinson's disease (PD) and Huntington's disease (HD), Pin1 is a pro-apoptotic factor in the process of neuronal degeneration, and high levels of Pin1 expression have been found in the brain tissue of patients 75-78. In other studies, downregulation of Pin1 expression was found to increase the likelihood of developing Alzheimer's disease (AD), and low expression of Pin1 was found in patients with AD 74, 78-80.

### Alzheimer's disease

**Figure 2:**
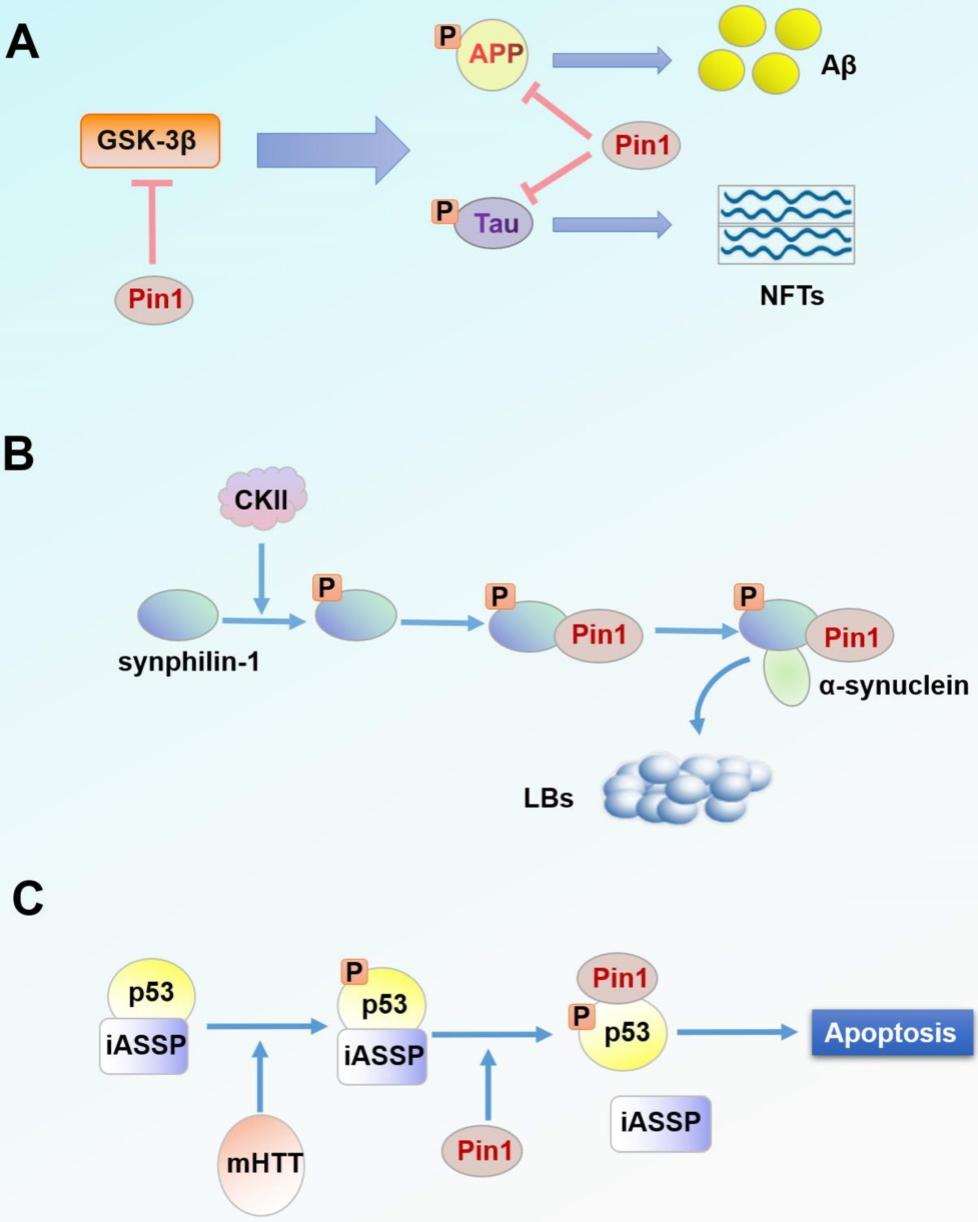
** Pin1 in the pathogenesis of neurodegenerative diseases. (A)** Accumulation of NFTs and Aβ is one of the pathogenic factors of AD. NFTs and Aβ are products of Tau and APP processing, respectively. Pin1 inhibits hyperphosphorylation of Tau protein and APP processing and suppresses upstream GSK-3β activity. **(B)** LBs are a characteristic protein polymer of PD. α-synuclein is the main component of LBs. Pin1 binds synphilin-1 phosphorylated by CKII and regulates its interaction with α-synuclein, thereby co-locating with α-synuclein intracellularly. **(C)** Pin1 binds and regulates p53 phosphorylated by mHTT. Subsequently, p53 is separated from the apoptosis inhibitor iASSP and is cascade activated, thus inducing neuronal apoptosis.

Increased deposition of plaques and intracellular neurofibrillary tangles (NFTs) are the main mechanisms of AD pathogenesis. NFTs are microtubule aggregations produced by hyperphosphorylation of Tau protein 74. Extracellular plaques are primarily composed of aggregates of amyloid-β-peptides (Aβ) derived from increased APP processing 74, 81, 82. In the neuronal cells of patients with AD, Pin1 is usually underexpressed and exhibits a negative correlation with degeneration of neuronal fibers 83. Pin1 catalyzes the conformational switch of GSK-3β-mediated phosphorylated Tau proteins from the dysfunctional cis structure to the functional trans structure, thus degrading Tau proteins 84-86. Additionally, Pin1 catalyzes phosphorylation of APP Thr^668^-Pro from the cis to trans isomer and also transforms APP processing to healthy non-amyloid formation 84. Pin1 can also directly inhibit activation of GSK-3β by binding to the phosphorylated Thr^330^-Pro motif of GSK-3β and catalyzing its isomerization 84, 87. Evidence suggests that overexpression of Pin1 in mature neurons can prevent neurodegeneration caused by Tau hyperphosphorylation 79. In general, events that decrease expression of Pin1 in the brain increase the likelihood of AD 88 **(Figure 2A).**

### Parkinson's disease

Lewy bodies (LBs) are the characteristic protein aggregates in tissues of PD. LBs are mainly composed of α-synuclein 89, 90, which is an unfolded protein in the natural state but can be induced to form an insoluble α-synuclein aggregate in the pathological state 91, 92. Synphilin-1 is a protein that can interact with α-synuclein; this interaction plays a very important role in the formation of LBs 93-95. Co-expression of α-synuclein and synphilin-1 causes the formation of debris inclusion bodies in the cytoplasm 93, 96. From immunohistochemical analysis of the brain tissue of patients with PD, Pin1 was found to be expressed in 50-60% of LBs and was co-located with α-synuclein in inclusion bodies 75. Due to the absence of a pSer/Thr-Pro motif in α-synuclein, Pin1 cannot bind to free α-synuclein but affects α-synuclein through indirect effects 75, 97. Under the phosphorylation mediated by casein kinase II (CKII), Pin1 binds to phosphorylated synphilin-1 through Ser^211^-Pro and Ser^215^-Pro motifs, thus indirectly interacting with α-synuclein 75. Overexpression of Pin1 could inhibit degradation of α-synuclein, enhance the half-life and insolubility of α-synuclein, and contribute to the formation of debris inclusion bodies of α-synuclein 75 **(Figure 2B).** Therefore, it can be speculated that inhibitors targeting Pin1 may alleviate the process of PD.

### Huntington's disease

HD is a neurodegenerative disease caused by repeated amplification of the gene encoding CAG in huntingtin protein (HTT) 98. The mutant huntingtin protein (mHTT) forms an endonuclear inclusion by misfolding and aggregating 99, 100. mHTT is toxic, and its aggregation causes glial proliferation of astrocytes and selective loss of striatal neurons 101, 102. mHTT can also cause DNA damage in neurons (DDR) 103, 104, which is a significant pathological feature of HD. Studies have found that p53 mediates this cytotoxicity in HD cells and transgenic animal models, while p53 inhibitors block this process 105. mHTT promotes phosphorylation of p53 at Ser^46^ through HIPK2 and PKCδ, making it a target for Pin1 binding and regulation 76. Pin1-mediated p53 isolates from the apoptosis-inhibiting factor iASPP, thus promoting the activation cascade of p53 in striatal neurons and increasing neuronal apoptosis 76, 106 **(Figure 2C).** Conversely, when Pin1 is silenced, p53 binds to iASPP regardless of mHTT expression and p53 fails to induce apoptosis, thereby preventing mHTT-dependent neurodegeneration 76. Pin1 is also associated with DDR in the regulation of DNA double-strand fracture repair 107. In one study, DNA damage signal intensity in Pin1-knockout mice was significantly reduced by 20% compared with that of the wild-type HD mouse model 77. However, another study revealed that Pin1 is a negative regulator of mHTT aggregation and that Pin1 overexpression reduces mHTT aggregates in HEK293 cells 108. Nevertheless, experimental results from human neuronal cells and HD mice suggest that Pin1 is a potential therapeutic target for HD treatment.

## Pin1 and viral infection

Viruses are common pathogens that cause infectious diseases. When a virus invades a host, the host activates its own immune system to fight or clear the infection 109. But there are proteins in the host that help the virus reduce resistance from the host or promote the viral infection process. Some studies have found that Pin1 is one of these proteins and is closely related to viral infections 20, 110-119.

### HIV

**Figure 3:**
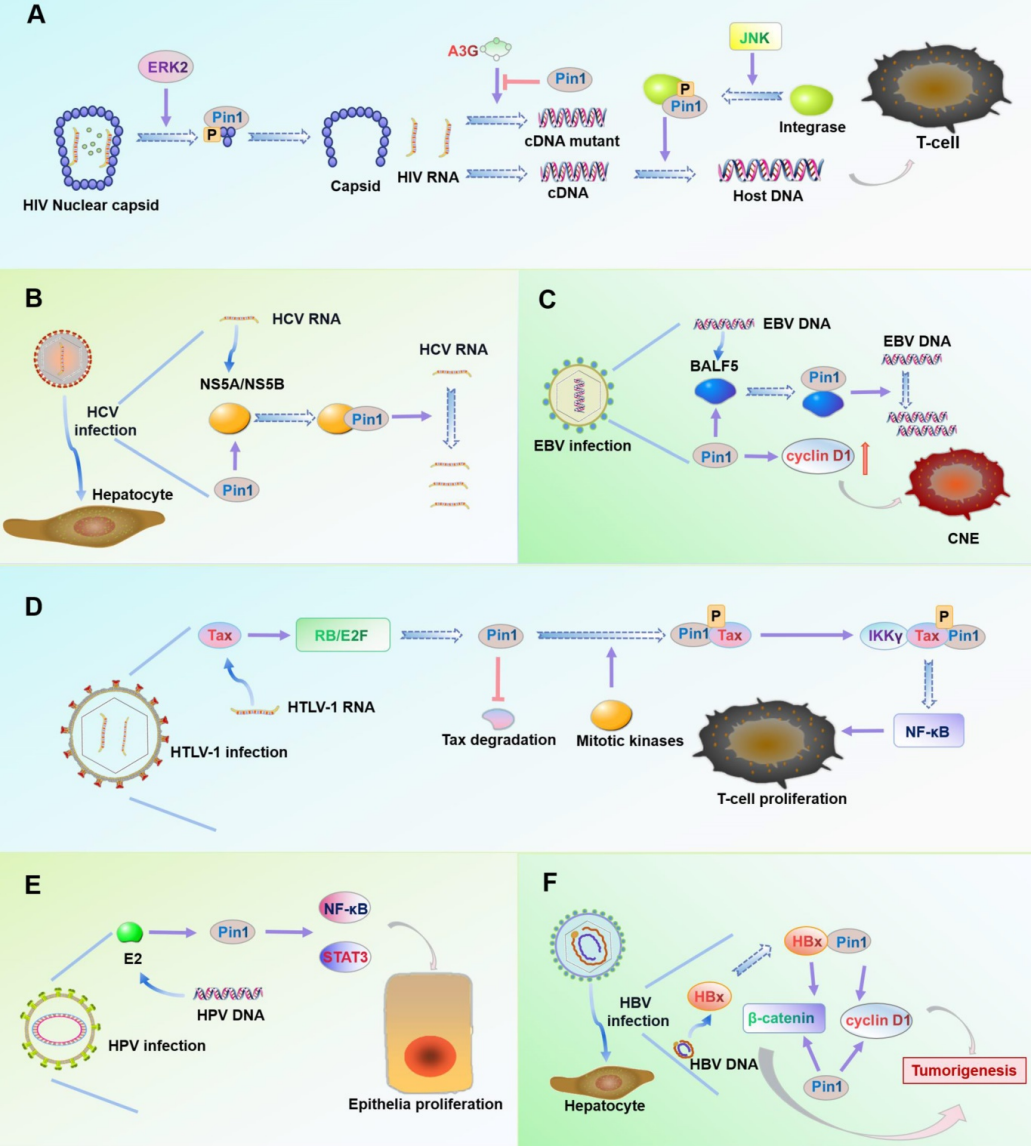
** Roles of Pin1 in virus infection. (A)** In HIV, the Ser^16^-Pro^17^ motif of the capsid protein is phosphorylated by ERK2. Pin1 specifically binds the motif and rearranges the capsid structure to release HIV RNA. Pin1 inhibits expression of catalytic polypeptide A3G to prevent incorrect coding of HIV cDNA during reverse transcription. Pin1 binds to the Ser^57^-Pro motif of integrase after its phosphorylation by the cellular kinase JNK, thus enhancing the stability of the integrase and promoting integration of HIV cDNA into the host cell DNA. **(B)** The viral protein NS5A/NS5B contains multiple phosphorylated Ser/Thr-Pro motifs. Interaction of overexpressed Pin1 with NS5A/NS5B increases HCV RNA replication and enhances HCV infection. **(C)** BALF5 is a key enzyme that regulates EBV DNA replication. Pin1 binds to the Thr^178^-Pro motif of BALF5 and actively regulates EBV DNA replication by regulating the conformation of the enzyme. Pin1 can also promote proliferation of EBV-infected nasopharyngeal carcinoma cells by upregulating expression of cyclin D1. **(D)** Viral oncoprotein Tax plays an important role in cell proliferation and viral replication. In HTLV-1-infected cells, Tax activates the RB/E2F pathway to increase expression of Pin1, which maintains the stability of Tax. Pin1 binds to the Ser^160^-Pro motif of Tax after its phosphorylation by mitotic kinase, which enhances the ability of Tax to directly bind IKKγ, activate NF-κB signaling, and finally promote cell proliferation and tumor occurrence. **(E)** In HPV-infected cells, overexpression of Pin1 causes NF-κB nuclear retention and activation of STAT3. Viral protein E2 can target and enhance the activity of Pin1 to increase the likelihood of cancer caused by HPV infection. **(F)** The viral protein HBx is a trans-activator of liver cancer. HBx activates cyclin D1 and the signaling pathway Wnt/β-catenin. Pin1 binds to the Ser^41^-Pro motif of HBx, which stabilizes the activity of HBx and induces overexpression of cyclin D1 and β-catenin, thus promoting liver cancer in HBV infection.

Acquired immunodeficiency syndrome (AIDS) is caused by human immunodeficiency virus (HIV) infection 120. Host protein Pin1 promotes HIV infection by mediating three key processes in the HIV replication cycle 121-123 **(Figure 3A).**

First, the HIV core relies on Pin1 to remove capsid proteins: The HIV core is composed of ribonucleic acid (RNA) molecules and capsid protein. When HIV infects a host, it must remove the capsid and release RNA for subsequent reverse transcription, replication, and other processes 124. The extracellular signal-regulated kinase 2 (ERK2) specifically phosphorylates Ser^16^-Pro^17^ residues on capsid proteins 124. Pin1 then binds to the phosphorylated Ser^16^-Pro^17^ motif, which rearranges the structure of the capsid protein and removes the capsid from the HIV core 125.

Second, Pin1 facilitates reverse transcription of the HIV genome: Reverse transcription of the HIV genome is an important step in the life cycle of HIV, as DNA produced by reverse transcription can be incorporated into the host genome 126. Host protein A3G (APOBEC3G) induces mutations in DNA during reverse transcription, which limits HIV replication 127. But Pin1 downregulates A3G expression and prevents A3G from entering HIV 128. HIV infection increases phosphorylation of Pin1 at Ser^16^ and enhances the inhibitory effect of Pin1 on A3G 111, 128.

Third, Pin1 helps integrate HIV cDNA into the host DNA: HIV needs to integrate its cDNA into the host genome to reliably transcribe its progeny RNA 129. The cellular kinase JNK phosphorylates the pSer^57^ motif of HIV integrase 130. Pin1 then binds to the phosphorylated pSer^57^-Pro motif to activate and stabilize HIV integrase activity, which helps it insert the HIV cDNA into the host genome 130, 131.

### HCV

Hepatitis C virus (HCV) is the main pathogen of chronic hepatitis and hepatocellular carcinoma (HCC). HCV is an enveloped RNA virus 132. The replication process of HCV depends mainly on the host cell cycle and requires participation of host proteins 133. Pin1 has been shown to be a necessary cytokine for HCV replication and can increase HCV infection 114. Overexpression of Pin1 increases intracellular HCV RNA and intracellular viral protein NS5A 114. HCV proteins NS5A and NS5B contain phosphorylated Ser/Thr-Pro motifs and Pin1 specifically interacts with NS5A and NS5B in immunoprecipitation experiments. NS5B can also increase expression of Pin1 114, 115. In general, host protein Pin1 may be utilized to increase HCV replication and infection **(Figure 3B).**

### EBV

Epstein-Barr virus (EBV) infection is associated with Burkitt lymphoma and production of T-cell malignancies 134. BALF5, the EBV DNA polymerase subunit, is a key enzyme that affects replication during EBV cleavage 135. Pin1 has been shown to be an important factor in regulating EBV replication. BALF5 interacts with Pin1 in a phosphorylation-dependent manner at Thr^178^-Pro of the BALF5 subunit. In one study, Pin1 knockdown by shRNA significantly inhibited EBV replication 119. Another study showed that Pin1 is overexpressed in all EBV-associated nasopharyngeal carcinoma (NPC) cells, xenografts, and primary tumors 20. Overexpression of Pin1 induces tumor cell growth by promoting production of cyclin D1 and activating the MAPK/JNK pathway **(Figure 3C).** The Pin1 inhibitor Juglone has been shown to inhibit growth of nasopharyngeal carcinoma cells and induce their apoptosis 20.

### HTLV-1

Human T-cell leukemia virus type 1 (HTLV-1) is the pathogen that causes adult T-cell leukemia (ATL) 136. The oncoprotein Tax encoded by HTLV-1 plays an important role in cell proliferation, viral gene replication, transformation, and tumor generation 137, 138. Expression of Tax may cause overexpression of Pin1 in ATL 116, 118. In cells infected by HTLV-1, Tax activates the E2F/RB pathway to increase transcription and expression of Pin1. Pin1 binds to the Tax phosphorylation motif pSer^160^-Pro in the presence of mitotic kinases. Pin1-regulated phosphorylated Tax then interacts with IKKγ to promote NF-κB activation 118, 139. The activity of NF-κB plays an important role in cell transformation, cell proliferation and cancer development 140. Pin1 can also inhibit both ubiquitination and lysosomal degradation of Tax, thus promoting its stability 116 **(Figure 3D).**

### HR-HPV

High-risk human papillomavirus (HR-HPV) is closely related to cervical cancer, with HPV16 being the most common subtype 141. E2 protein is a factor that regulates viral replication and transcription and can be a marker for early HPV infection 142. Cancers caused by HR-HPV infection may be associated with activation of transcription factors NF-κB and STAT3 143. Overexpression of Pin1 in cervical cancer can increase nuclear retention of NF-κB and promote transactivation of STAT3, further promoting the occurrence of cancer 144-146. In one study, increased Pin1 expression in E2-transfected HEK293 cells was found to be not significant (0.3-fold increase); however, E2 was found to enhance the activity of Pin1 117. This data indicates that E2 regulates activation of transcription factors NF-κB and STAT3 by targeting the activity of Pin1 117, which further effects cancer progression **(Figure 3E).**

### HBV

Hepatitis B virus (HBV) is a common pathogen in hepatocellular carcinoma (HCC) and HBV-encoded protein HBx is a trans-activator of liver cancer 147. A study found that overexpression of Pin1 was most common in HBV-related HCC, and the majority of cases showed co-expression of Pin1 and HBx 113. HBx contains two phosphorylated Ser-Pro motifs that are potential targets for Pin1 148, 149. Pin1 binds to the phosphorylated Ser^41^-Pro motif of HBx, which increases HBx stability and transactivation 113, 149. HBx activates the oncogenic transcription factor cyclin D1 and the β-catenin signaling pathway associated with oncogenesis 150. Overexpression of Pin1 not only increases expression of cyclin D1 but also promotes intracellular accumulation of β-catenin in the Wnt/β-catenin signaling pathway 151, 152. These two aspects can increase expression of oncogenes and promote occurrence of HCC in HBV infection **(Figure 3F).**

In summary, Pin1 promotes most viral infectious diseases by two broad mechanisms: (1) Pin1 is directly involved in the life cycle of the virus to promote viral infection. For example, Pin1 is involved in core exuviation, reverse transcription, and integration of the virus in HIV infection 111. In HCV infection, Pin1 is involved in viral RNA replication 114. Pin1 is also involved in viral DNA replication in EBV infection 119. (2) Pin1 enhances the stability and production of oncogenic proteins. For example, Pin1 increases the stability of Tax in HTLV-1 infection and mediates Tax transactivation of NF-κB factor 116, 118. In HBV infection, Pin1 stabilizes the oncoprotein HBx and increases expression of oncogenic proteins cyclin D1 and catenin 112, 113. In HPV infection, Pin1 is involved in increased activation of transcription factors STAT3 and NF-κB 117. Pin1 also increases expression of the oncogenic protein cyclin D1 in EBV infection 20. Tanaka *et al.* also found that dipentamethylene thiuram monosulfide, a specific inhibitor of Pin1, inhibited feline coronavirus (FCoV) replication 153, suggesting that targeting Pin1 may provide new insights for antiviral therapy.

## Conclusions and future directions

In summary, our review illustrates the potential roles of Pin1 in several common diseases. Due to the overexpression of Pin1 in tumor tissues and its role in promoting tumor progression, drugs currently under development for targeting Pin1, including natural products, chemical compounds, and peptide drugs, are mainly focused on cancer treatment. Although Pin1 inhibitors have shown tumor suppressive effects in cell lines, animal models, and even clinical trials, some inhibitors reveal a Pin1-independent mechanism and the side effects have yet to be clarified. For example, Pin1 inhibitor KPT-6566 may exert anti-cancer effects through at least two simultaneously acting mechanisms: inhibition of Pin1 and ROS production 31. API-1 shows significant anti-HCC activity, but its low water solubility and *in vivo* bioavailability limit its clinical application 154. Researchers have also identified some compounds or peptides such as PEPTIDE 155 and benzothiophene 156, 157 that suppress Pin1 activity at nanomolar concentrations but are inactive in cell-based assays because of their poor membrane permeability. One potential countermeasure is to increase the membrane permeability of these compounds by optimizing their structure or looking for corresponding derivatives. Research on the application of Pin1 inhibitors and agonists in other related diseases is limited, and more detailed investigations need to be carried out for therapeutic potential, especially in diseases such as viral infection and AD in which the role of Pin1 is relatively clear. Studies on the upstream regulatory signals and downstream targets of Pin1 can also provide ideas for expanding treatment strategies for the above-mentioned diseases.

## Figures and Tables

**Table 1:** Oncogenic proteins/growth-promoting regulators and tumor suppressors/growth-inhibitory regulators as Pin1 substrates

Substrate	Function	Activity of substrate	Refs
AIB1	Oncogenic protein	↑	158
AKT	Oncogenic protein	↑	159
BCL2	Oncogenic protein	↑	160
JUN	Oncogenic protein	↑	23
COX2	Oncogenic protein	↑	161
FOS	Oncogenic protein	↑	162
FOXM1	Oncogenic protein	↑	163
HER2	Oncogenic protein	↑	164
MYC	Oncogenic protein	↑	165
Survivin	Oncogenic protein	↑	46
Tax	Oncogenic protein	↑	118
XBP1	Oncogenic protein	↑	166
AR	Growth-promoting regulator	↑	167
CDC25	Growth-promoting regulator	↑	168
Cep55	Growth-promoting regulator	↑	169
MYB	Growth-promoting regulator	↑	170
Cyclin D1	Growth-promoting regulator	↑	23
ER	Growth-promoting regulator	↑	164
FAK	Growth-promoting regulator	↑	171
HBx	Growth-promoting regulator	↑	113
HIF1	Growth-promoting regulator	↑	172
HSF1	Growth-promoting regulator	↑	173
IRAK1	Growth-promoting regulator	↑	174
MCL1	Growth-promoting regulator	↑	175
Nanog	Growth-promoting regulator	↑	176
NF-κB	Growth-promoting regulator	↑	177
NOTCH1	Growth-promoting regulator	↑	178
NOTCH3	Growth-promoting regulator	↑	179
NUR77	Growth-promoting regulator	↑	180
OCT4	Growth-promoting regulator	↑	181
p47phox	Growth-promoting regulator	↑	182
p53M	Growth-promoting regulator	↑	183
PGK1	Growth-promoting regulator	↑	115
PKM2	Growth-promoting regulator	↑	184
PLK	Growth-promoting regulator	↑	185
PML-RARα	Growth-promoting regulator	↑	186
PTP	Growth-promoting regulator	↑	187
PTP-PEST	Growth-promoting regulator	↑	171
RAB2A	Growth-promoting regulator	↑	188
RAF1	Growth-promoting regulator	↑	189
RSK2	Growth-promoting regulator	↑	190
S642	Growth-promoting regulator	↑	189
S6K	Growth-promoting regulator	↑	191
Separase	Growth-promoting regulator	↑	192
SEPT9	Growth-promoting regulator	↑	193
BRD4	Growth-promoting regulator	↑	45
STAT3	Growth-promoting regulator	↑	146
v-Rel	Growth-promoting regulator	↑	194
β-catenin	Growth-promoting regulator	↑	151
BAX	Tumor suppressor	↓	47
CDK10	Tumor suppressor	↓	195
CtIP	Tumor suppressor	↓	107
DAXX	Tumor suppressor	↓	196
FADD	Tumor suppressor	↓	197
FBXW7	Tumor suppressor	↓	198
FOXO4	Tumor suppressor	↓	199
IRF3	Tumor suppressor	↓	200
KLF10	Tumor suppressor	↓	201
PML	Tumor suppressor	↓	202
pRb	Tumor suppressor	↓	203
RARα	Tumor suppressor	↓	186
RUNX3	Tumor suppressor	↓	204
SMRT	Tumor suppressor	↓	205
AMPK	Growth-inhibitory regulator	↓	206
ATR	Growth-inhibitory regulator	↓	207
AUF1	Growth-inhibitory regulator	↓	208
BTK	Growth-inhibitory regulator	↓	209
Che1	Growth-inhibitory regulator	↓	210
GRK2	Growth-inhibitory regulator	↓	211
p27	Growth-inhibitory regulator	↓	212
PIP4K	Growth-inhibitory regulator	↓	213
RBBP8	Growth-inhibitory regulator	↓	107
SMAD	Growth-inhibitory regulator	↓	55
Smad3	Growth-inhibitory regulator	↓	214
SUV39H1	Growth-inhibitory regulator	↓	215
TRF1	Growth-inhibitory regulator	↓	216
XPO5	Growth-inhibitory regulator	↓	217

AIB1: amplified in breast cancer 1; AKT: the serine/threonine protein kinase B; AMPK: AMP-activated protein kinase; AR: androgen receptor; ATR: ataxia telangiectasia and Rad3 related; BCL2: B-cell lymphoma 2; CDC25: cell division cycle 25; CDK10: cyclin-dependent kinase 10; Cep55: centrosome protein 55; COX2: cyclooxygenase-2; ER: estrogen receptor; FAK: focal adhesion kinase; FBXW7 : F-box and WD40 repeat domain containing-7; FOXM1: forkhead box M1; FOXO4: forkhead box O4; HBX: hepatitis B virus X-protein; HER2: human epidermal growth factor receptor 2; HIF-1: hypoxia-inducible transcription factor-1; HSF1: heat shock transcription factor 1; IRAK1: interleukin-1 receptor-associated kinase 1; IRF3: interferon-regulatory factor 3; KLF10: kruppel-like factor 10; MCL1: myeloid cell leukemia-1; NF-κB: nuclear factor kappa-light-chain- enhancer of activated B cells; OCT4: octamer 4; PGK1: phosphoglycerate kinase 1; PKM2: pyruvate kinase M2; PLK: polo-like kinase; PML: promyelocytic leukemia protein; PML-RARα: promyelocytic leukemia- retinoic acid receptor alpha; pRb: retinoblastoma protein; PTP: protein tyrosine phosphatase; RARα: retinoic acid receptor alpha; RSK2: ribosomal protein S6 kinase 2; RUNX3: runt-related transcription factors 3; S6K: S6 kinase; SMRT: silencing mediator for retinoic acid and thyroid hormone receptor; STAT3: signal transducer and activator of transcription 3; XBP1: X-box-binding protein 1.
